# Geopolymer Foams Loaded with Diatomite/Paraffin Granules for Enhanced Thermal Energy Storage

**DOI:** 10.3390/ma18194512

**Published:** 2025-09-28

**Authors:** Agnieszka Przybek

**Affiliations:** 1 CUT Doctoral School, Cracow University of Technology, Warszawska 24, 31-155 Cracow, Poland; agnieszka.przybek@pk.edu.pl; 2 Faculty of Material Engineering and Physics, Cracow University of Technology, Jana Pawła II 37, 31-864 Cracow, Poland; 3 Interdisciplinary Center for Circular Economy, Cracow University of Technology, Warszawska 24, 31-155 Cracow, Poland

**Keywords:** geopolymer foams, thermal insulation composites, diatomite granules containing paraffin, phase change energy storage materials, advanced multifunctional building materials

## Abstract

This paper presents the development and characteristics of geopolymer foams modified with paraffin-based phase change materials (PCMs) encapsulated in diatomite. The aim was to increase both the thermal insulation and heat storage capacity of the foams while maintaining sufficient mechanical strength for construction applications. Eleven variants of composites with different PCM fractions (5–10% by mass) and grain sizes (<1.6 mm to >2.5 mm) were synthesized and tested. The inclusion of PCM encapsulated in diatomite modified the porous structure: the total porosity increased from 6.6% in the reference sample to 19.6% for the 1.6–1.8 mm_10% wt. variant, with pore diameters ranging from ~4 to 280 µm. Thermal conductivity (λ) ranged between 0.090–0.129 W/m·K, with the lowest values observed for composites 2.0–2.5 mm_5–10% wt. (≈0.090–0.091 W/m·K), which also showed high thermal resistance (R ≈ 0.287–0.289 m^2^·K/W). The specific heat (Cp) increased from 1.28 kJ/kg·K (reference value) to a maximum value of 1.87 kJ/kg·K for the 2.0–2.5 mm_10% mass variant, confirming the effective energy storage capacity of PCM-modified foams. Mechanical tests showed compressive strength values in the range of 0.7–3.1 MPa. The best structural performance was obtained for the 1.6–1.8 mm_10% wt. variant (3.1 MPa), albeit with a higher λ (≈0.129 W/m·K), illustrating the classic trade-off between porosity-based insulation and mechanical strength. SEM microstructural analysis and mercury porosimetry confirmed the presence of mesopores, which determine both thermal and mechanical properties. The results show that medium-sized PCM fractions (1.6–2.0 mm) with moderate content (≈10% by weight) offer the most favorable compromise between insulation and strength, while thicker fractions (2.0–2.5 mm) maximize thermal energy storage capacity. These findings confirm the possibility of incorporating natural PCMs into geopolymer foams to create multifunctional materials for sustainable and energy-efficient building applications. A unique contribution to this work is the use of diatomite as a natural PCM carrier, ensuring stability, compatibility, and environmental friendliness compared to conventional encapsulation methods.

## 1. Introduction

The modern economy faces an enormous challenge related to the need to reduce primary energy consumption and greenhouse gas emissions, while at the same time experiencing a systematic increase in energy demand resulting from civilizational development, urbanization, and improvements in the standard of living of societies [1,2,3]. According to the International Energy Agency (IEA), the construction and building operation sector accounts for approximately 30% of total global energy consumption and nearly 26% of carbon dioxide emissions, making it one of the main areas requiring modernization and innovation [4]. It is particularly important to reduce the energy used for heating, cooling, and air conditioning, as these processes generate the greatest energy losses and have a key impact on user comfort [5].

One of the priorities of energy and climate policy is to improve the energy efficiency of buildings through the use of new technologies and materials that not only reduce heat loss but also enable active energy flow management. This research focuses, among other things, on solutions that enable the accumulation of excess heat when it is available and its release when needed, which allows for the equalization of internal temperature fluctuations and a reduction in external energy consumption [6]. For this reason, materials capable of storing thermal energy and releasing it in a controlled manner are considered one of the most promising groups of innovative solutions in sustainable construction, which can contribute to the achievement of climate goals and improve user comfort [7].

Thermal energy storage can be achieved in several ways, which differ in both mechanism and efficiency. Explicit storage involves increasing the temperature of a material without changing its state of aggregation. This solution is technically simple and widely used (e.g., in water or brine tanks), but its efficiency is limited by the relatively low heat capacity of the materials and temperature fluctuations during energy release [8]. Chemical storage, on the other hand, uses reversible chemical reactions that allow very large amounts of energy to be stored over the long term. Although this technology is extremely promising, its implementation in construction practice is limited by high costs, process complexity, and safety issues [9].

In this context, we are talking about a phase change with latent heat storage, which occurs during the melting and solidification of a substance. Phase change materials (PCMs) accumulate energy during the transition from a solid to a liquid state (melting) and release it in the reverse process, i.e., from a liquid to a solid state (solidification), at an almost constant transition temperature. As a result, PCMs have a much higher energy storage density compared to overt storage (e.g., in water tanks), while ensuring thermal stability [10]. This type of phase change is physical, not chemical, which means that the material does not undergo any chemical composition changes or degradation during the melting-solidification cycles. This is particularly important in building applications, as PCMs can act as a thermal buffer, absorbing excess heat during the day when the internal temperature rises and then gradually releasing it at night, which limits temperature fluctuations and reduces the load on heating and cooling systems [11].

For this reason, phase change materials are gaining increasing interest in the context of energy-efficient construction and passive thermal energy management systems. The integration of PCMs into building elements such as walls, ceilings, and plaster allows for an improvement in the energy balance of buildings by reducing the energy demand for heating and cooling [12]. These materials operate passively, i.e., without the need for additional mechanical devices, which makes them both effective and economically advantageous in the long term [13].

The use of PCMs directly translates into improved thermal comfort for users, as it stabilizes the internal temperature of the building, limiting sudden fluctuations throughout the day. This makes it possible to reduce dependence on active air conditioning or heating systems, which not only lowers operating costs but also reduces primary energy consumption [14,15,16]. This effect is crucial in the context of global efforts to decarbonize the economy, as lower demand for energy from conventional sources is associated with a reduction in greenhouse gas emissions, primarily carbon dioxide [17,18].

As a result, phase change materials are currently considered one of the most promising elements supporting the development of sustainable construction. Their implementation is in line with European Union policy and International Energy Agency guidelines on improving energy efficiency in the construction sector [19]. In combination with other solutions, such as smart energy management systems, renewable energy sources, and modern insulation, PCMs can play an important role in creating low-carbon and comfortable living and commercial spaces [20].

The most commonly used phase change materials in construction applications include paraffins, i.e., mixtures of linear aliphatic hydrocarbons with varying chain lengths. They are widely used due to a number of beneficial properties [21]. First of all, they are characterized by a relatively high phase change enthalpy, which allows for the accumulation of amounts of thermal energy per unit of mass [22]. They also exhibit good chemical stability and resistance to degradation processes during repeated melting-solidification cycles, and are non-toxic and safe for users [23]. Another important advantage of paraffins is their wide melting range, which can be adjusted by selecting appropriate hydrocarbon fractions, allowing them to be adapted to various operating conditions, including applications in energy-efficient construction, air conditioning, and passive heat storage systems [24].

Despite their numerous advantages, paraffins also have disadvantages that limit their direct use in building structures. The most serious limitation is their very low thermal conductivity, which hinders effective energy exchange and prolongs the charging and discharging time of the material [25]. The second key problem is its tendency to leak in its liquid state, leading to material loss and reduced composite durability. As a result, paraffins used on their own do not meet the practical requirements for long-term and repeatable use in building components [26,27].

For this reason, intensive research is being conducted on methods to improve the functionality of paraffin as a PCM. One approach is micro- or macro-encapsulation, i.e., enclosing the phase change material in polymer, ceramic, or composite coatings that protect against leakage and increase the heat exchange surface area. Another equally promising method is to immobilize paraffin in porous mineral carrier structures [28,29]. Thanks to the sorption capabilities of such materials, it is possible for the liquid to penetrate the pores and stabilize in the matrix, which largely eliminates the problem of migration during phase transitions. An additional advantage of this approach is the improvement of the thermal conductivity of the system by introducing a solid phase with favorable heat transport properties into the composite [30].

One of the most commonly studied porous materials used as a matrix for immobilizing PCMs is diatomite (diatomaceous earth). It is a natural sedimentary rock of organic origin, formed as a result of the accumulation of the shells of single-celled algae—diatoms. These shells are composed mainly of amorphous silica (SiO_2_·nH_2_O), which gives diatomite specific physicochemical properties [31]. It is characterized by a very large specific surface area, high porosity, and low bulk density, which in practice means excellent sorption capacity for liquids and gases. The porous structure of diatomite allows paraffin to easily penetrate and remain inside the micropores, which effectively prevents its migration during phase change cycles. This makes diatomite an extremely effective matrix for stabilizing PCMs and limiting leaks [32].

Another advantage of diatomite is its widespread availability and low cost of extraction, as it occurs in numerous deposits around the world and is used in many industries, including filtration, insulation materials, and as an adsorbent. It is a non-toxic, chemically stable, and environmentally friendly material, which further increases its attractiveness in terms of application, especially in the construction sector, which requires the use of safe and durable raw materials [33,34].

The use of diatomite as a carrier for paraffin leads to the creation of composite phase change materials (CPCMs), in which paraffin is permanently embedded in a porous structure. This solution not only eliminates the problem of leakage but also improves the thermal and functional stability of the composite under conditions of repeated melting-solidification cycles [35,36]. In addition, the siliceous nature of diatomite improves the thermal conductivity of the entire system, which increases the rate of heat exchange between the PCM and the environment. As a result, paraffin- and diatomite-based CPCMs are considered to be among the most promising materials for use in energy-efficient building systems and other areas requiring stable and repeatable thermal energy storage [37,38].

The literature describes two main approaches to solving problems related to the use of paraffins as phase change materials (PCMs): limiting leakage through micro- or macroencapsulation or immobilization in porous carriers, and improving thermal conductivity by introducing highly conductive phases such as graphite, activated carbon, carbon nanotubes, or metallic additives [29,39]. Comparative studies have shown that immobilizing paraffin in porous structures (e.g., diatomite, zeolites, silica) effectively minimizes liquid phase migration even during multiple transition cycles, while maintaining high heat storage capacity [31,40]. On the other hand, the use of conductive additives systematically increases the thermal conductivity coefficient λ, shortening the heat charging and discharging time, although sometimes at the expense of increased conduction in the solid state [41,42]. The best results are usually achieved through hybrid strategies, combining PCM immobilization in a porous carrier (leak control) with dispersed conductive additives (improved heat exchange) [43]. Research results suggest that optimal parameters are achieved with a moderate proportion of a highly porous carrier and an addition of ~1–5 wt.% graphite or nanofillers [44]. An alternative approach is to modify the surface of the carrier (e.g., through chemical functionalization), which enhances the wettability and adhesion of the liquid phase, thereby reducing the need for large amounts of conductive additives [45]. In the case of integration with geopolymer matrices, especially in the form of foams, the use of immobilized PCMs (e.g., diatomite/paraffin) allows for the minimization of leaks and cyclic stabilization while increasing the heat capacity of the composite and maintaining favorable thermal insulation properties [46,47].

Parallel to the development of variable-phase materials technology, geopolymer materials are being intensively researched. In recent years, they have been gaining importance as an environmentally friendly alternative to traditional cement binders. Geopolymers are inorganic materials obtained through the polycondensation of alkali-activated aluminosilicates. This process leads to the formation of a three-dimensional aluminosilicate network with a structure resembling natural zeolite minerals [48,49]. Their most important advantages include a low carbon footprint compared to Portland cement, high mechanical strength, resistance to chemical agents, and the possibility of precisely shaping their properties by selecting raw materials (e.g., metakaolin, fly ash, metallurgical slag) and synthesis conditions. An additional advantage of geopolymers is the possibility of using industrial waste as raw materials, which not only reduces production costs but also supports the circular economy [50,51].

A particularly important feature of geopolymers is the possibility of using industrial and construction waste as raw materials, which not only reduces production costs but also supports the circular economy. The literature describes various sources of waste used in geopolymers, including red mud [52], recycled glass waste [53,54], and brick and construction waste [54,55]. The use of such raw materials not only reduces the amount of waste sent to landfills but also reduces the carbon footprint of building materials, which is important in the context of sustainable construction. At the same time, the literature indicates that the introduction of lightweight materials such as perlite or zeolite allows for a significant reduction in the density of geopolymers [54,56]. Lowering the density improves thermal insulation properties, which is beneficial in energy-efficient construction and in structures requiring lightweight fillings. The use of perlite and zeolite affects the development of pores and the microstructure of the material, helping to reduce thermal conductivity and increase thermal resistance [56]. In addition, research on microencapsulated phase change materials (PCMs) in lightweight concretes and geopolymers indicates the possibility of producing composites that combine lightness, insulation, and heat storage capacity [52,54]. However, there are still significant research gaps in the context of foam geopolymers with PCM additives, in which the effects of PCM particle size and content, porosity, and pore distribution on mechanical and thermal properties are analyzed simultaneously. There is also a lack of systematic studies comparing different waste sources in the geopolymer matrix in terms of their compatibility with PCMs and heat storage efficiency [55,56]. There is a clear need for research combining the advantages of lightweight geopolymers and PCMs to design low-density, high-insulation, and increased heat capacity foam composites, while utilizing industrial and construction waste.

A special group of these materials is geopolymer foams, which are produced by foaming a geopolymer matrix using gas-forming additives or by mechanically introducing air bubbles. This process gives them low density and a porous structure, which provides them with a number of unique functional properties. These foams are characterized by high thermal insulation, fire resistance, good dimensional stability, and the possibility of reducing the weight of structural elements [57,58]. Thanks to these features, they can be used in lightweight, energy-efficient building components such as insulation panels, wall blocks, and thermal insulation layers in composite partitions [59].

Furthermore, geopolymer foams have sound absorption properties, are non-flammable, and resistant to high temperatures, which increases their potential for improving fire safety in buildings. Due to their properties and low environmental impact, they are considered one of the most promising construction materials in the field of sustainable building [60,61]. Their natural porosity and cellular structure also make them ideal candidates for integration with phase change materials, opening up new possibilities for the creation of hybrid materials with both insulating and accumulating properties [40].

The combination of the storage properties of phase change materials (PCMs) with the porous structure of geopolymer foams opens up new perspectives in the design of innovative building materials with enhanced thermal energy management capabilities [62,63]. The integration of these two solutions allows for the creation of a material that simultaneously functions as a traditional insulating layer and an active heat storage medium. This makes it possible not only to reduce energy losses but also to store energy during periods of surplus and gradually release it during periods of shortage, which helps to stabilize the internal microclimate of buildings on a daily and seasonal basis [64].

The introduction of a PCM into the structure of geopolymer foams provides a synergistic effect—the porous, lightweight, and fire-resistant geopolymer matrix provides a stable environment for the phase change material, while the PCM gives the foams additional storage functionality. Such hybrid composites can improve the energy balance of buildings, contributing to a reduction in external energy demand and greenhouse gas emissions. This solution is therefore in line with the concept of sustainable construction and the implementation of climate neutrality strategies [54,65].

At the same time, the use of diatomite as a PCM carrier plays a key role in ensuring the durability and functionality of the entire system. The porous structure of diatomite allows for effective immobilization of paraffin, minimizing the risk of leakage during repeated melting-solidification cycles [66]. This results in greater thermal and mechanical stability of the material, which is an important condition for its practical use in construction. In addition, the presence of silica in the structure of diatomite improves thermal conductivity, which translates into more efficient energy exchange between the PCM and the environment [67].

As a result, composites based on geopolymer foams, paraffin, and diatomite can be considered a new generation of functional materials, combining insulating, fire-resistant, and heat-accumulating properties. Such solutions pave the way for the development of energy-efficient and intelligent building components that respond to the challenges of today’s energy economy.

Previous research on building composites with phase change materials has focused mainly on traditional substrates such as cement mortars, plasters, and gypsum composites. Although these solutions allow for a certain increase in thermal energy storage capacity, they have limitations related to thermal conductivity, PCM cycle life, and the possibility of introducing amounts of phase change material without compromising mechanical properties. Much less attention has been paid to the use of geopolymer foams as a matrix for PCMs, even though their unique properties—low density, porous structure, fire and chemical resistance—make them ideal for this type of application. The literature points to the growing potential of using phase change materials (PCMs) in geopolymer foams to improve their thermal insulation properties and heat storage capacity. Studies on polyurethane foams coated with geopolymer with PCM capsules have shown a significant improvement in heat energy accumulation while maintaining the structural integrity of the material [68]. Similar effects have been observed in foamed geopolymer structures based on fly ash, where the addition of PCMs has reduced thermal conductivity while increasing heat capacity [69]. Reviews of innovative geopolymer foams with natural fibers and PCMs emphasize the importance of both the appropriate selection of PCM fractions and fiber reinforcements to achieve optimal thermal insulation and mechanical properties [70]. These results indicate a significant research gap in the field of geopolymer foams with PCMs, particularly about the optimization of microstructure and PCM proportions in lightweight composites. This research gap points to innovation potential: combining geopolymer foams with paraffin immobilized in porous mineral carriers, such as diatomite, could create a new class of hybrid materials combining thermal insulation, structural durability, and high thermal energy storage capacity. This approach has not been widely described in the literature, and its implementation could bring benefits both in terms of the energy efficiency of buildings and the development of functional materials with low environmental impact. This work aimed to develop and comprehensively characterize geopolymer foams modified with a composite phase-change material based on paraffin enclosed in diatomite. Particular emphasis was placed on assessing the ability of such materials to accumulate thermal energy and their mechanical and thermal properties relevant in the context of energy-efficient construction. The presented research results show the potential for creating a new generation of building materials with increased functionality, combining insulation, heat accumulation, and structural durability. Furthermore, hybrid PCM-geopolymer foam solutions are in line with the strategy of a low-carbon economy and sustainable development, offering a practical tool for reducing primary energy consumption and greenhouse gas emissions in construction. Emphasizing these aspects makes the work an innovative contribution to the field of functional materials and energy-efficient construction technologies. Despite extensive research on phase change materials, there are still significant gaps in our knowledge. One of these is the lack of clear analyses of the impact of PCM particle size on the performance properties of composites. The effectiveness of paraffin immobilization in diatomite compared to microencapsulation, which offers high retention control but involves greater complexity and costs, also remains insufficiently researched. In addition, there is a lack of studies that systematically assess the possibilities of integrating immobilized PCMs with geopolymer foams, especially in terms of their thermal and mechanical properties. Against this background, this work is innovative in that it focuses on analyzing the effectiveness of using diatomite as a PCM carrier and its integration with a porous geopolymer structure. This approach not only reduces leakage but also highlights the potential for the practical use of PCM-geopolymer composites in construction applications.

Despite numerous studies on geopolymers and alkali-activated lightweight composites, there is a lack of comprehensive analyses of materials that combine insulating properties with heat storage capacity while maintaining adequate mechanical strength. Research on the introduction of paraffinic phase change materials (PCMs) enclosed in natural carriers, such as diatomite in geopolymer foams, which could enable the creation of multifunctional materials for energy-efficient construction, is particularly limited. Most previous work has focused either on thermal properties or mechanical strength, without investigating the complementary effect of PCM addition on microstructure, porosity, and simultaneous changes in thermal and mechanical parameters. This work is unique in that it systematically investigates the effect of natural PCM granules with controlled fraction and content on the properties of geopolymer foams, enabling simultaneous improvement of thermal insulation and heat storage capacity while maintaining structural strength. Furthermore, the work provides a detailed analysis of the relationship between the size and quantity of PCM granules and microstructure, thermal conductivity, thermal resistance, heat capacity, and compressive strength, allowing the optimal compromise between insulation and strength to be identified. Such a comprehensive approach, integrating microstructural, thermal, and mechanical aspects, has not yet been fully presented in the literature, which emphasizes the innovative nature of this work and its importance for the development of highly functional and energy-efficient building materials.

## 2. Materials and Methods

### 2.1. Structural Elements of Geopolymer Foams

The basic raw material used in the research was class F fly ash, obtained as a by-product of hard coal combustion in the power boilers of the Skawina Power Plant (CEZ Skawina S.A., Skawina, Poland). In order to improve the mechanical properties of the composite and give it volume stability, an inorganic filler in the form of fine-grained quartz sand from the Świętochłowice Sand Plant (Świętochłowice, Poland) was also used. Thanks to its high mineralogical purity and appropriate granulation, this sand acted as a thickening agent, limiting the shrinkage of the composite and increasing the volume density of the geopolymer foams. Another component of the mixture was Górkal 70 high-alumina cement (Górka Cement Sp. z o.o., Trzebinia, Poland), which was introduced as a hydraulic additive. Its presence contributed to the intensification of the setting and hardening processes, especially in conditions of elevated temperature and increased humidity. The aluminum oxides contained in it enabled additional hydration reactions, which strengthened the emerging structure of the material and improved its resistance to environmental factors. In order to obtain a porous and lightweight structure, ash microspheres supplied by TERMO-REX S.A. (Jaworzno, Poland) were introduced into the matrix. These spherical particles, with low density and high mechanical strength, served as a lightweight filler, which contributed to reducing the volume weight of the foams while increasing their thermal insulation properties. The presence of microspheres also promoted an even distribution of pores, which was important for the functional properties of the material. To ensure the stability of the foaming process and obtain a uniform distribution of pores in the matrix, an organic surfactant, syringaldehyde (Merck, Darmstadt Germany), was added to the mixture. This substance acted as a surfactant, facilitating the formation and maintenance of gas bubbles during the foaming reaction, while at the same time limiting their coalescence in the initial bonding phase. This resulted in a more regular and closed-cell pore structure, which had a direct impact on the thermal insulation and mechanical properties of the geopolymer foams obtained. The chemical composition of fly ash, sand, cement, and microspheres was analyzed using X-ray fluorescence (XRF) with a SHIMADZU EDX-7200 device (SHIMADZU Europa GmbH, Duisburg, Germany), and the results are presented in Table 1. In addition, particle size distribution analysis was performed using the Anton-Paar PSA 1190LD laser diffractometer (Anton Paar GmbH, Graz, Austria), and the results are summarized in Table 2 [71].

### 2.2. Diatomite/Paraffin Granules as a Phase Change Material

**Figure 1:**
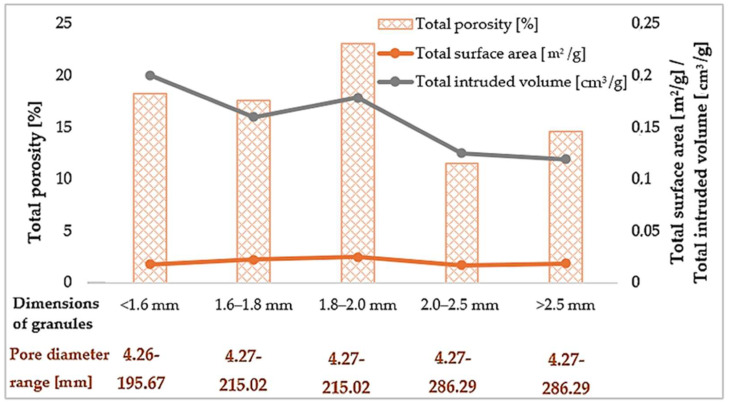
Porosity and pore structure parameters of diatomite-paraffin granules.

**Figure 2:**
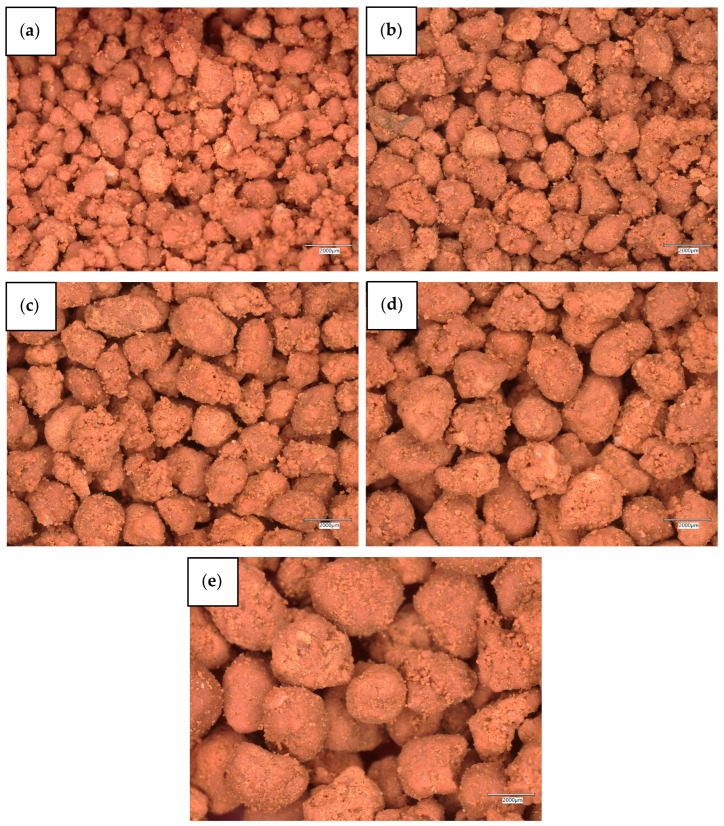
Macroscopic photographs of diatomite-paraffin granules taken at 20× magnification: (**a**) <1.6 mm, (**b**) 1.6–1.8 mm, (**c**) 1.8–2.0 mm, (**d**) 2.0–2.5 mm, (**e**) >2.5 mm.

Diatomaceous earth powder calcined at 850 °C was used for the impregnation process. The use of this processed raw material was due to its increased absorbency of petroleum and paraffin substances, which was confirmed in sorption studies described in other works by the authors [72,73]. The first stage of the paraffin macroencapsulation process in the diatomite structure was to saturate the mineral carrier with a phase-change substance. For this purpose, paraffin oil produced by FLEXOL (FLEXOL, Iława, Poland) was used for this purpose, selected due to its highest absorbency by diatomite, confirmed in the author’s previous studies [72]. Paraffin used as a phase change material has a phase transition range of −30 to −3 °C. Its thermal conductivity coefficient ranges from 0.1 to 0.3 W/m·K, which confirms its insulating properties. The material has a relatively high ignition temperature of 260 °C, which emphasizes its safety in use, and its density is 0.862 g/cm^3^. This substance was used in liquid form. The impregnation process was carried out by adding 69.7% by weight of paraffin to diatomite powder. This value corresponds to the maximum theoretical content of the substance that can be adsorbed by the porous structure of diatomite, calculated on the basis of its density, the density of paraffin, and the absorbency parameters determined experimentally. After combining the ingredients, the mixture was left in laboratory conditions (ambient temperature 23 °C, relative humidity 40–60%) for about 4 h, which allowed for full penetration of the pores and stable bonding of the paraffin with the carrier. The next step was to form diatomite granules containing paraffin. For this purpose, a GT-1 disc granulator (Atest sp. z o.o., Kielce, Poland) was used. The granulation process was carried out at a disc angle of 30° and a rotational speed of 35 rpm, which, based on preliminary tests, ensured the most uniform and stable granule formation. The parameters included a range of disc inclination adjustment from 0° to 60° and a rotational speed from 0 to 50 rpm. A composition of binders with different characteristics was used as a binder. The basic component was a solution of R-137 sodium water glass mixed with demineralized water in a 50/50 weight ratio. In addition, methylcellulose adhesive reinforced with synthetic resin and natural polysaccharide components in the demineralized form of flours and starches (wheat, potato, corn, coconut, and rice) was introduced. The organic additives selected in this way acted as binding agents, helping to increase the mechanical strength of the granules and improve their structural integrity. The binders were added in three equal proportions: water glass, methylcellulose glue, and organic additives after the initial granulation process using a disc granulator. The impermeability of liquid PCM is ensured by filling the pores of diatomite with paraffin during impregnation. Paraffin, penetrating the pores of the mineral carrier, is retained in the diatomite structure thanks to the capillary effect and adhesion to the pore walls. No additional protective coating was used in the process—the stability and lack of PCM leakage result solely from the physical retention of paraffin in the pores of the carrier. Binding additives, such as water-soluble soda glass, methylcellulose, and natural polysaccharides, serve to mechanically reinforce the granules, increasing their integrity and resistance to damage during handling and granulometric classification. The final stage of the process was the classification of the obtained granules into specific size fractions. For this purpose, an ANALYSETTE 3 PRO vibrating shaker (MERAZET S.A., Poznań, Poland) was used. Due to its application, the material was separated into five granulometric groups: <1.6 mm, 1.6–1.8 mm, 1.8–2.0 mm, 2.0–2.5 mm, and >2.5 mm. This division enabled further comparative analyses of both the physicochemical properties and the performance parameters of the granules. The results of the porosity tests of the obtained granules are presented in Figure 1, which includes quantitative data on total porosity [%], pore diameter range [µm], specific surface area [m^2^/g], and total intruded volume [cm^3^/g] for individual particle size fractions. These parameters allow a detailed evaluation of the structural capacity of diatomite granules to immobilize paraffin within their porous structure. The thermal characteristics are summarized in Table 3, providing values of thermal conductivity (λ, [W/m·K]), thermal resistance (R, [m^2^·K/W]), and specific heat capacity (Cp, [kJ/kg·K]) measured for different fractions. These data directly reflect the insulating performance and heat storage potential of the diatomite–paraffin composites, enabling identification of the most effective fraction for use in geopolymer foam modification. Macroscopic photographs illustrating the morphology and granulation diversity are presented in Figure 2.

Based on the tests conducted on the porosity and thermal properties of diatomite-paraffin granules, clear correlations can be observed between the porous structure and the heat accumulation and conductivity capacities. The highest total porosity was found in granules with a fraction of 1.8–2.0 mm (23.22%), which was also associated with the largest specific surface area (0.0251 m^2^/g) and a relatively large intruded volume. This explains their greatest capacity to accumulate paraffin in the carrier structure. The lowest porosity was found for the 2.0–2.5 mm fraction (11.59%), which also had the lowest intruded volume (0.1260 cm^3^/g), indicating a more compact and less absorbent structure. Although the range of pore diameters in individual fractions did not differ (from approximately 4 to 200–280 µm), in the case of larger fractions, a tendency towards a wider pore size range was observed, which, however, did not translate into higher total porosity. These relationships are also reflected in the thermal parameters. The lowest thermal conductivity coefficient was recorded for the 1.8–2.0 mm fraction (0.10562 W/m·K), which correlates with their high porosity and the presence of a large number of closed pores that reduce thermal conductivity. Fractions with lower porosity, such as <1.6 mm and >2.5 mm, had higher λ values (0.12070 and 0.11838 W/m·K, respectively) and thus poorer insulating properties. Thermal resistance (R) reached its highest values for the smallest and largest fractions (<1.6 mm—0.2579 m^2^·K/W and >2.5 mm—0.2485 m^2^·K/W), although a comparable level was also recorded for the 1.8–2.0 mm fraction (0.2378 m^2^·K/W), which indicates that both porosity and granule geometry affect heat transport. The specific heat in the tested temperature range was between 1.16–1.55 kJ/kg·K. The lowest value was recorded for the 2.0–2.5 mm fraction (1.160 kJ/kg·K), which confirms its limited capacity to store thermal energy. In turn, the highest Cp values were recorded for the >2.5 mm (1.550 kJ/kg·K) and 1.8–2.0 mm (1.548 kJ/kg·K) fractions, which indicates their favorable accumulation properties. The most optimal properties in terms of thermal insulation and accumulation capacity are exhibited by granules with a fraction of 1.8–2.0 mm. They combine high porosity, large specific surface area, low thermal conductivity, and high heat capacity, which makes them the most effective carrier in the macroencapsulation of phase change materials. Fractions of 2.0–2.5 mm, due to their lowest porosity and weaker thermal parameters, should be considered the least favorable. The smallest and largest fractions exhibit intermediate parameters, combining moderate thermal conductivity with good heat capacity, but do not achieve as favorable a balance of properties as the 1.8–2.0 mm fraction.

Macroscopic photographs (20×) show diatomite-paraffin granules in five fractions: <1.6 mm, 1.6–1.8 mm, 1.8–2.0 mm, 2.0–2.5 mm, and >2.5 mm. Visual observations show that the morphology of the granules changes with increasing fraction size—the smallest (<1.6 mm) are compact and have a relatively smooth surface, while the medium-sized ones (1.6–2.0 mm) have a more developed and rough texture. The largest fractions (>2.5 mm) show a more heterogeneous shape, with visible irregularities and larger surface defects. Macroscopic photographs do not allow for the assessment of actual internal porosity (which was determined using quantitative methods and summarized in Table 1), but they do illustrate well the differences in the morphology and compactness of the granules of individual fractions.

### 2.3. Synthesis and Formation of Geopolymer Foams

**Figure 3:**
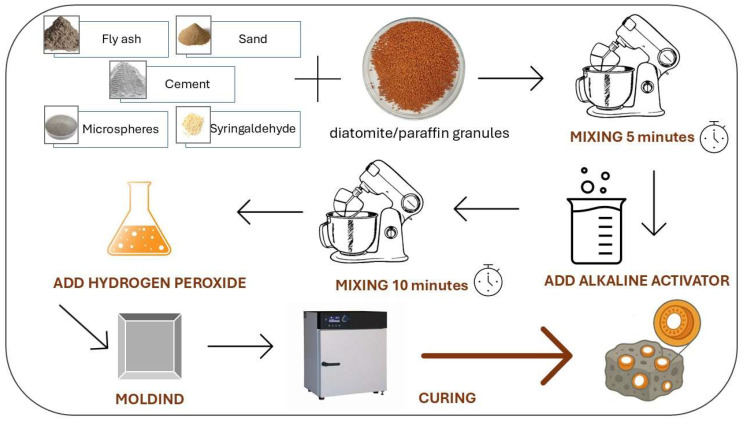
Scheme of the production of geopolymer foams with diatomite/paraffin granules.

Geopolymer foams were produced using an automated mixing process carried out with a GEOLAB M/LMB-s laboratory mixer (Warsaw, Poland), designed in compliance with standards for mortar and concrete preparation. The procedure started with a dry mixing stage, during which class F fly ash—used as the main mineral component—was placed in the mixer chamber together with diatomite–paraffin granules and additional agents supporting foam stability and pore structure development (as described in Section 2.1). This stage lasted 5 min at 50 rpm, enabling preliminary homogenization and uniform distribution of ingredients. Subsequently, a liquid alkaline activator was introduced, consisting of a 10 M NaOH solution (prepared by dissolving technical-grade granules, purity >99%, PCC Rokita SA, Brzeg Dolny, Poland, in distilled water) and an R-145 sodium silicate solution (SiO_2_/Na_2_O = 2.5, density ≈ 1.45 g/cm^3^, Zakłady Chemiczne ANSER, Wiskitki, Poland). These two components were combined in a 1:2.5 mass ratio (NaOH: water glass), forming a stable, highly reactive activator of the geopolymerization process. After the solution was added, mixing continued for 10 min, producing a uniform plastic mass with rheological properties suitable for molding and foaming. To create a porous structure dominated by closed pores, 35% hydrogen peroxide was used as a foaming agent. Following its addition, mixing proceeded for another 2 min, ensuring uniform dispersion and initiating oxygen release, which generated the pore system. The fresh mass was then transferred into prepared laboratory molds, covered with protective foil to enable free foam expansion. The specimens were cured in an SLW 750 chamber dryer (POL-EKO Perfect-Environment, Wodzisław Śląski, Poland) at 60 °C for 24 h—a critical stage for geopolymerization and the initial strength development of the material. Afterward, the samples were removed from the molds and conditioned for 28 days under laboratory conditions (room temperature, ~50% RH), allowing full structural consolidation and the development of mechanical and physicochemical properties. The final specimens were plate-shaped, measuring 20 × 20 × 2.5 cm. Their detailed composition and labeling are summarized in Table 4. Figure 3 shows a diagram of the production of geopolymer foams with diatomite/paraffin granules.

### 2.4. Porosity of Geopolymer Foams with PCM Based on Diatomite and Paraffin

The porosity of the obtained geopolymer foams was tested using a PoreMaster 33 mercury porosimeter (Anton-Paar, Graz, Austria), operating in the low-pressure range. This device allows measurements to be taken in the range from 0.2 to 50 psi, which enables the analysis of pores of varying sizes, from nanometers (approx. 6.4 nm) to macropores with diameters of up to 1100 µm. The principle of operation of the porosimeter is based on the penetration of liquid mercury into the capillary system of the porous material under increasing pressure, which allows both the diameters and the total volume of the pores of the tested material to be determined. The PoreMaster 33 is an advanced measuring tool that enables detailed characterization of the porous structure of building and functional materials, including geopolymer foams. Using this method, it is possible to determine parameters such as pore size distribution, intruded volume, and total porosity, which allows for a comprehensive determination of the microstructural properties of the material. As part of this study, all 11 variants of the prepared geopolymer composites were analyzed. This allowed for a comparison of their porous structure and provided a complete picture of the porosity distribution, which is necessary for further interpretation of the physicochemical and functional properties of the foams.

### 2.5. Compressive Strength of Geopolymer Foams with PCM Based on Diatomite and Paraffin

Compressive strength testing of the geopolymer foams was conducted using a universal testing machine, MTS Criterion 43 (Eden Prairie, MN, USA), equipped with TestSuites 1.0 software and a load capacity of up to 30 kN. The apparatus is specifically designed for accurate mechanical testing of construction materials, including cement mortars and geopolymer concretes, enabling precise load control and real-time data acquisition. The procedure followed the guidelines of the European standard PN-EN 12390-3:2019-07 (Testing of concrete—Part 3: Compressive strength of test specimens) [74], while the geometry and dimensions of the samples complied with PN-EN 12390-1:2013-03 [75]. Cubic specimens with dimensions of 25 × 25 × 25 mm were used. Each sample was positioned in the load chamber to ensure that the compressed surfaces were aligned perpendicularly to the applied force. The reduced sample size was chosen to maintain consistency with thermal conductivity tests, for which the specimens had a thickness of 25 mm. This approach allowed for comparable evaluation of both mechanical and thermal properties of the same material. The load was applied progressively and uniformly until specimen failure, enabling determination of the maximum destructive force (Fc). The compressive strength (Rc) was then calculated based on Fc using Equation (1). This methodology ensured result reproducibility and provided a reliable basis for comparing the mechanical properties of different geopolymer foam variants, which is essential for assessing their potential in structural and functional applications.

(1) $$R_{c}=\frac{F_{c}}{A}[MPa]$$

where

Rc—compressive strength [MPa],
A—sample cross-sectional area [mm^2^],
Fc—maximum load [N].

### 2.6. Thermal Parameters of Geopolymer Foams with PCM Based on Diatomite and Paraffin

The thermal properties of the foamed geopolymer materials were evaluated using a Netzsch HFM 446 Lambda heat flow meter (Selb, Germany), an advanced plate apparatus designed for precise determination of insulation parameters in building materials, including geopolymer composites. The device operates on the two-plate method (hot plate and cold plate) and complies with international standards ASTM C1784 [76], ASTM C518 [77], ISO 8301 [78], and EN 12664 [79]. Its wide measurement range (0.007–2.0 W/m·K) enables analysis of both lightweight foams and denser composites, while high accuracy (±1–2%), repeatability (±0.25%), and reproducibility (±0.5%) ensure reliable results. Measurement stability was further supported by precise temperature regulation via Peltier modules. Thermal conductivity (λ) and thermal resistance (R) were determined in the temperature range of 0–20 °C, representative of real operating conditions of insulation materials in construction. In addition, the specific heat capacity (Cp) was measured between 27.5 and 32.5 °C, together with bulk density, providing a comprehensive thermophysical characterization of the foams. Specimens were prepared as plates fitted to the dimensions of the measuring chamber to guarantee proper contact with the plates and uniform temperature distribution during testing. Their mass was measured using a RADWAG PS 200/2000.R2 analytical balance (Radom, Poland; accuracy 0.01 g), and their dimensions were determined with a caliper (resolution 0.01 mm). From these data, λ, R, Cp, and density were calculated, enabling a full evaluation of the material’s functionality as a modern, low-emission insulating material with thermal storage capacity. The results provide a basis for further assessment of the foams’ applicability in lightweight construction systems requiring both effective insulation and heat accumulation.

### 2.7. Macroscopic Images of Geopolymer Foams with PCM Based on Diatomite and Paraffin

Macroscopic observations of the foamed geopolymer materials were carried out using a Keyence VHX-7000 digital optical microscope (KEYENCE INTERNATIONAL (BELGIUM) NV/SA, Mechelen, Belgium), an advanced system for high-resolution surface imaging and documentation of engineering materials. The purpose of the study was to assess the porous structure of the samples, which plays a key role in their thermal insulation performance. Prior to imaging, the sample surfaces were cleaned and mechanically leveled to enable precise focusing and to minimize the influence of surface irregularities. Owing to its advanced optical system and automatic focus adjustment, the VHX-7000 allowed for the acquisition of clear, detailed images without the need for further specimen preparation. All macroscopic images were recorded in reflected light mode using ring illumination and the HDR function, which automatically optimized exposure settings to enhance contrast and detail. Additionally, the integrated Depth Composition function enabled accurate three-dimensional reconstruction of the sample surfaces, effectively capturing the complex pore morphology and irregular topography of the foamed geopolymers.

### 2.8. Microscopic Images of Geopolymer Foams with PCM Based on Diatomite and Paraffin

The microstructure of the geopolymer foams was examined using a JEOL IT200 scanning electron microscope (JEOL, Akishima, Tokyo, Japan). Small fragments of the material were prepared as test specimens, carefully cleaned of dust and fine particles generated during separation. To preserve the structure of the PCM-containing materials, the fragments were dried at 40 °C until constant weight was achieved. For SEM analysis, the samples were mounted on carbon discs attached to metal stubs in the microscope holder. EM-Tec C33 carbon adhesive was applied to improve sample adhesion and enhance electrical conductivity. To further ensure imaging quality, the specimens were sputter-coated with a thin gold layer using a DII-29030SCTR Smart Coater vacuum system (JEOL Ltd., Peabody, MA, USA). This preparation enabled the acquisition of high-resolution SEM images, allowing detailed visualization of the foam microstructure.

## 3. Results

### 3.1. Porosity of Geopolymer Foams with PCM Based on Diatomite and Paraffin

Table 5 presents a summary of key parameters characterizing the porous structure of geopolymer foams modified with diatomite-paraffin granules acting as a phase change material (PCM). All 11 variants of the developed composites were analyzed, which allowed for a comprehensive assessment of the impact of the additive on the structural properties of the material. The list includes basic indicators describing porosity, including the total closed porosity in the sample, which plays an important role in the process of heat accumulation and thermal conductivity of insulating materials. In addition, the range of pore sizes is presented, allowing for the determination of the morphological diversity of the internal structure of the foams, as well as the specific surface area—a parameter directly related to the possibility of sorption, heat transport, and interaction with the environment. The values of the total volume of mercury intruded during porosimetric analysis are also included, which allows for a quantitative assessment of the available pore spaces and the degree of their filling. The summary is a starting point for further interpretation, allowing the porosity properties to be linked to the thermophysical and mechanical characteristics of the geopolymer materials tested. This provides a more complete understanding of the relationship between the porous structure and the effectiveness of foams as composite thermal insulators with increased energy storage capabilities.

Analysis of the results presented in Table 5 indicates that the addition of diatomite–paraffin granules (PCM) affects the development of porosity in geopolymer foams compared to the reference sample (F.A.). The reference material was characterized by low total porosity of 6.6% and a relatively small volume of intruded mercury (0.2642 cm^3^/g), while having a well-developed specific surface area (0.0574 m^2^/g), which suggests a limited number of pores but a relatively active internal surface. In the modified samples, the porosity increase reached up to +198% compared to the reference (1.6–1.8 mm, 10 wt.%—19.64%), while the intruded mercury volume rose by nearly +77% (0.4676 cm^3^/g). A similarly high rise in porosity was recorded for the 1.8–2.0 mm, 5 wt.% fraction (+179%) with an increase of +59% in intruded volume, confirming that medium grain size promotes the development of an extensive pore structure. Fine fractions (<1.6 mm) caused moderate but stable increases in porosity by +104–134% and in intruded volume by +43–64%, which confirms their effective impact on microstructural modification. In contrast, the coarser fractions (2.0–2.5 mm and >2.5 mm) produced more varied results. For the 2.0–2.5 mm, 5 wt.% sample, porosity decreased by −18.5% and the intruded volume dropped by −32%, suggesting insufficient dispersion of large granules and a more compact structure. However, at a higher content (10 wt.%), porosity increased by +86% and intruded volume by +13%, while the >2.5 mm, 10 wt.% fraction showed a porosity increase of +116% and intruded volume increase of +59%, indicating the formation of additional voids around larger particles. It is worth noting that the increase in porosity and intruded volume was accompanied by a downward trend in specific surface area: reductions of −7% to −46% compared to the reference (e.g., 0.0451 m^2^/g for the 1.8–2.0 mm, 10 wt.% variant). This phenomenon is characteristic of materials with developed macroporosity, in which larger pores dominate, reducing the total active surface area but increasing the total void volume. Overall, the use of PCM leads to a modification of the pore structure of geopolymer foams. The most beneficial effects are obtained for medium fractions (1.6–2.0 mm, 5–10 wt.%), which achieve the largest relative increases in porosity and pore volume, indicating their strong potential for improving heat accumulation and exchange capacity. Medium PCM granules with sizes of 1.6–2.0 mm best support the development of the porous structure of geopolymers, achieving the greatest relative increase in total porosity and pore volume. Fine granules (<1.6 mm) caused a moderate but stable increase in porosity and pore volume, which indicates their effective but limited modification of the microstructure, while larger granules (>2.0 mm) showed more varied effects—at low content, insufficient dispersion and local compaction of the material occurred, and at higher mass, they created additional spaces around the particles. Medium granules combine the advantages of both approaches: they are large enough to stably form pores, but at the same time small enough not to cause local compaction of the structure. This results in optimal dispersion in the matrix, a developed pore structure with high relative increases in porosity (+179–198%) and pore volume (+59–77%), which promotes heat storage and exchange. The balance between macro- and micropores allows for significant heat capacity while maintaining an effective active surface area, even though the dominance of larger pores slightly reduces the total specific surface area. As a result, medium granules of 1.6–2.0 mm prove to be the most effective in improving the heat storage and thermal exchange capacity of the material.

### 3.2. Compressive Strength of Geopolymer Foams with PCM Based on Diatomite and Paraffin

**Figure 4:**
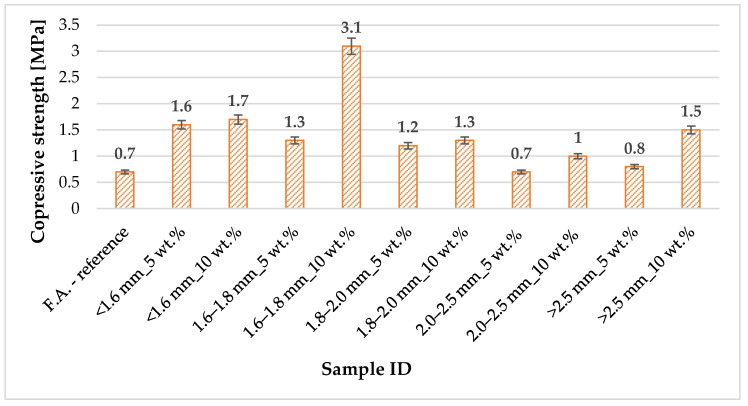
Compressive strength of geopolymer foams loaded with diatomite-paraffin granules.

Figure 4 summarizes the results of tests on the mechanical properties of geopolymer foams modified with diatomite-paraffin granules acting as a phase change material (PCM). The analysis focused primarily on compressive strength, which is a key parameter characterizing the resistance of materials to mechanical loads and allows their potential use in construction as structural and insulating elements to be assessed. The tests were carried out on prepared samples representing different variants of composites, taking into account the varied content and fraction of PCM granules. To ensure the reliability and repeatability of the results, each series of samples was subjected to five independent measurements, and then the average values were calculated and presented in the table. This approach not only eliminates possible measurement errors but also allows for a reliable assessment of the impact of PCM additives on the mechanical parameters of foams. The values listed in the table form the basis for further analysis of the relationship between the content of phase change material and the compressive strength of foams, which is particularly important in the context of their service life and long-term structural stability.

Analysis of the compressive strength results of geopolymer foams with the addition of diatomite-paraffin granules (PCM) shows a clear influence of both the size of the granules and their weight content on the mechanical properties of the materials. The reference value, corresponding to foam without PCM addition, was 0.7 MPa, which is the benchmark for assessing the changes introduced by the modification. The use of a fine fraction < 1.6 mm resulted in a noticeable increase in strength—both at 5% (1.6 MPa) and 10% (1.7 MPa) content, which suggests a beneficial effect of fine granules on the filling and stabilization of the porous structure. An even more pronounced effect is observed in the case of the 1.6–1.8 mm fraction, where a 10% content achieved the highest value among all the variants tested—3.1 MPa, which is more than four times higher than in the reference sample. This indicates that it is this combination of PCM granule size and concentration that provides the best reinforcement of the foam structure. In contrast, for the 1.8–2.0 mm fraction, the strength values obtained were lower, close to 1.2–1.3 MPa, which means a weaker reinforcement effect compared to finer fractions. Even weaker results were obtained when using granules with sizes of 2.0–2.5 mm, where the strength values were comparable or slightly higher than the reference values (0.7–1.0 MPa). A similar, moderate increase was observed for the largest granules > 2.5 mm, where the compressive strength reached 0.8 MPa at a 5% addition and 1.5 MPa at a 10% addition. The results clearly indicate that the key factor determining the strength of foams is not only the quantity, but above all, the fraction of PCM granules used. The most favorable mechanical properties were observed for the 1.6–1.8 mm fraction at 10% content, which suggests the existence of an optimal particle size that allows for even distribution in the geopolymer matrix and effective reinforcement of its structure. An analysis of the compressive strength results of geopolymer foams with the addition of diatomite-paraffin granules (PCM) indicates that both the size of the granules and their weight content in the material play a key role. The highest strength values were obtained for the 1.6–1.8 mm fraction with a 10% addition (3.1 MPa), which is more than four times higher than in the reference sample without PCM (0.7 MPa). Microstructurally, this effect can be explained by the uniform distribution of medium-sized granules in the geopolymer matrix, which promotes effective pore filling and cell structure stabilization. Granules of this size are small enough to penetrate the spaces between macropores, minimizing stress concentration and reducing local defects, yet large enough to form a stable ‘scaffold’ in the foam structure that aids load transfer. In the case of finer granules (<1.6 mm), a significant increase in strength is also observed, but the effect is smaller because particles that are too small do not form a distinct macropore scaffold, and their effect is mainly limited to modifying the microstructure of the pores. On the other hand, larger granules (>2.0 mm) introduced inhomogeneities in the pore distribution, which led to local compaction or discontinuities in the structure, resulting in a moderate improvement in strength. Consequently, it can be concluded that the pore size distribution and the optimal selection of granule fractions directly affect the mechanical reinforcement of the geopolymer matrix—medium granules provide a balance between macroporous stabilization and microstructure filling, resulting in the highest compressive strength while maintaining a favorable porous structure. In the context of the literature on insulation materials, the compressive strength of lightweight mineral and geopolymer foams typically ranges from 0.2 to 2.0 MPa, with materials with high porosity and good insulating properties often achieving values at the lower end of this range. Compared to these data, the composites tested show significantly improved mechanical properties, especially in the case of finer PCM fractions, combining relatively high strength with the preservation of insulating properties, which is a significant achievement in the context of designing multifunctional building materials.

### 3.3. Thermal Parameters of Geopolymer Foams with PCM Based on Diatomite and Paraffin

Table 6 summarizes the results of tests on the thermal properties of geopolymer foams modified with diatomite-paraffin granules acting as phase change materials (PCM). The analysis covered key parameters determining the thermal insulation and accumulation efficiency of these composites, i.e., the thermal conductivity coefficient (λ) and thermal resistance (R), which were determined in the temperature range corresponding to typical operating conditions for building materials, i.e., 0–20 °C. In addition, the specific heat (Cp), which is a measure of the material’s ability to accumulate thermal energy, was examined in the temperature range of 27.5–32.5 °C—a range that is particularly important from the point of view of the functioning of PCMs, in which phase transitions occur. In addition to thermal parameters, the density of composites was also analyzed, which is an important indicator describing the degree of compaction of the porous structure and its impact on thermal conductivity and accumulation capacity. Taking this parameter into account allows for a more complete correlation between the microstructural characteristics of the tested foams and their behavior under thermal load conditions, and also facilitates the interpretation of differences between individual material variants. All values presented in the table are average results obtained from two independent measurements, which increases the reliability of the data and reduces the impact of measurement errors. This approach allows for a more precise characterization of the tested foams, while also enabling the identification of relationships between porosity, density, and the ability to conduct and accumulate heat. By taking into account several physical, mechanical, and thermal parameters at the same time, this comparison provides a comprehensive basis for assessing the suitability of modified geopolymer foams as modern insulation materials with dual functionality—low thermal conductivity and increased heat storage capacity. It is particularly important to compare the λ and R values with the porosity and density parameters and to relate the Cp value to the proportion and size of PCM granules in the geopolymer matrix, which allows for a better understanding of the mechanism of improving the performance of these composites.

Analysis of the obtained results of the thermal properties and density of geopolymer foams with the addition of diatomite-paraffin granules (PCM) clearly shows the influence of both the granule content and fraction on the material properties. For the reference sample (without PCM addition), the density is 337.65 kg/m^3^, the thermal conductivity coefficient (λ) is 0.09248 W/m·K, and the thermal resistance (R) reaches 0.2831 m^2^·K/W. In most cases, the addition of PCM granules increases the density of the composites, as expected due to the introduction of an additional solid phase. The largest increase is observed for the 1.6–1.8 mm_10 wt.% variant, whose density rises to 550 kg/m^3^ (+62.9% compared to the reference). This is accompanied by a substantial increase in thermal conductivity (λ = 0.12883 W/m·K, +39.2%) and a decrease in thermal resistance (R = 0.2021 m^2^·K/W, −28.6%), indicating a reduction in insulating performance. This behavior can be attributed to the densification of the foam structure and a decrease in the fraction of pores, which are primarily responsible for lowering thermal conductivity. Interestingly, for samples containing 2.0–2.5 mm granules, the 5 wt.% variant shows a density close to the reference (339.20 kg/m^3^, +0.44%), while the thermal conductivity coefficient is slightly lower (λ = 0.09027 W/m·K, −2.39%). Consequently, the thermal resistance is the highest among all tested samples (R = 0.2888 m^2^·K/W, +1.91%), suggesting an optimal balance between porosity and PCM distribution within the geopolymer matrix. Specific heat capacity (Cp) values further highlight the added functionality of the PCM composites. Compared to the reference (1.280 kJ/kg·K), most variants exhibit higher Cp, reaching a maximum of 1.873 kJ/kg·K (+46.3%) for the 2.0–2.5 mm_10 wt.% sample. This indicates that the modified foams can store more thermal energy, which is advantageous for passive energy storage applications. Overall, PCM addition not only increases density but also modifies thermal properties. Variants with moderate density (e.g., 2.0–2.5 mm_5 wt.% and >2.5 mm_5 wt.%) provide the best compromise between low thermal conductivity and high heat storage capacity. Conversely, excessive densification, particularly with high content of finer PCM fractions, leads to reduced insulation performance. When comparing these results with cement-based PCM composites, which often exhibit thermal conductivity ranges from 0.2 to 0.6 W/m·K at higher densities and relatively low porosity, geopolymer PCM foams offer a significant advantage by combining low thermal conductivity with high porosity. High concentrations of fine granules, on the other hand, cause excessive compaction, increasing conductivity and reducing insulating properties, which shows that optimal granule size distribution and moderate PCM content are key to balancing insulation and heat storage capacity.

### 3.4. Macroscopic Images of Geopolymer Foams with PCM Based on Diatomite and Paraffin

**Figure 5:**
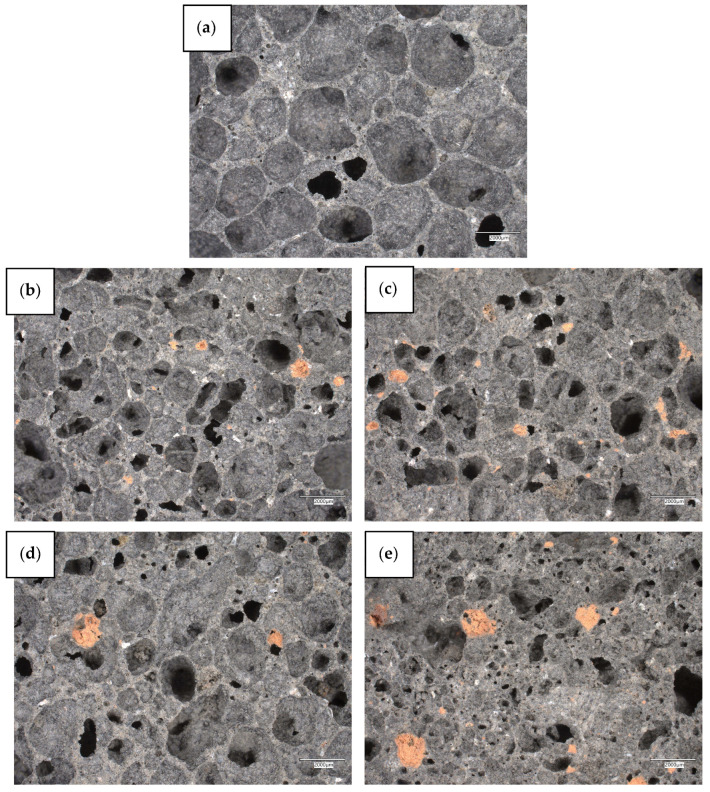

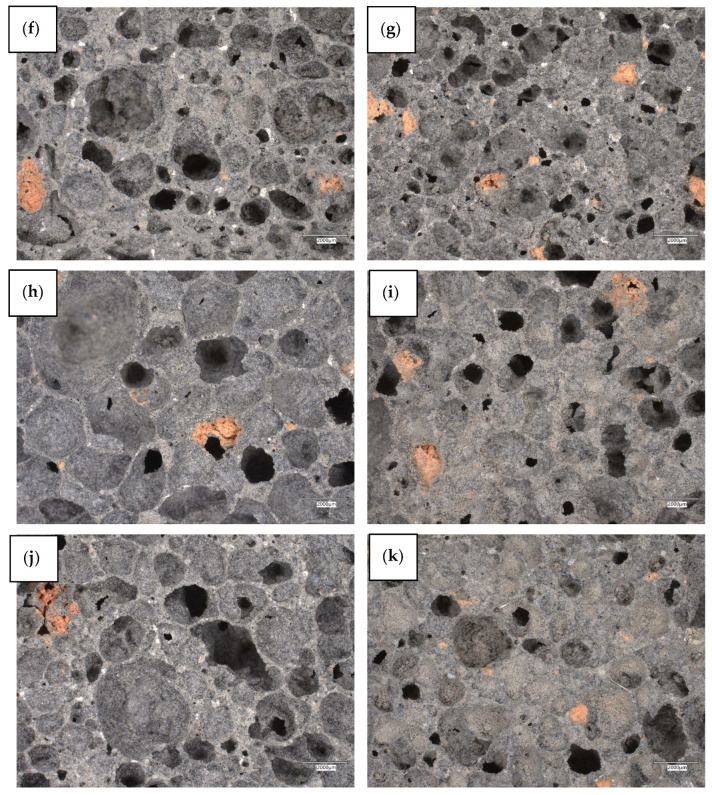
Macroscopic images of geopolymer composites with PCMs: (**a**) F.A.—reference, (**b**) <1.6 mm_5 wt.%, (**c**) <1.6 mm_10 wt.%, (**d**) 1.6–1.8 mm_5 wt.%, (**e**) 1.6–1.8 mm_10 wt.%, (**f**) 1.8–2.0 mm_5 wt.%, (**g**) 1.8–2.0 mm_10 wt.%, (**h**) 2.0–2.5 mm_5 wt.%, (**i**) 2.0–2.5 mm_10 wt.%, (**j**) >2.5 mm_5 wt.%, (**k**) >2.5 mm_10 wt.%.

Figure 5 shows macro photographs of the obtained geopolymer composites, which allow for visual assessment of their surface and overall structure. The images were taken at 20× magnification, which allows for the capture of morphological features invisible to the naked eye, such as pore distribution, matrix homogeneity, and the presence of diatomite-paraffin granules. This allows for a preliminary comparison of the quality of the obtained materials, as well as the identification of differences resulting from the modification used and the PCM content in the composite. Macrophotographs are therefore an important supplement to quantitative analyses, providing visual data that allow for a better understanding of the relationship between the microstructure and the physical and mechanical properties of geopolymer foams.

The macro photographs shown in Figure 5 reveal the surface structure of the geopolymer foams produced with the addition of diatomite-paraffin granules. All photos show numerous pores of various shapes and sizes, which is characteristic of foamed geopolymers. The pores are both closed and partially connected, which indicates a heterogeneous structure of the foams. The variants marked with letters from (b) to (k) show varying pore sizes—smaller PCM fractions generate numerous small pores, while larger fractions lead to the formation of larger air chambers, which can be observed in some variants. Most of the images show orange fragments corresponding to diatomite-paraffin granules, distributed relatively evenly, although in some areas, especially at higher PCM contents, slight agglomeration is observed. The geopolymer matrix, i.e., the gray phase, is cohesive and fills the spaces between the pores and PCM granules well, with no visible cracks or large defects, suggesting that the mixing and foaming process was effective. Analysis of structural dependencies on PCM fractions and content shows that smaller fractions (<1.6 mm) generate finer pores and a more homogeneous surface, which promotes mechanical stability and low thermal conductivity, while medium and larger fractions (1.6–2.5 mm and >2.5 mm) create larger pores and a more diverse structure, which may increase heat capacity but potentially reduce compressive strength. Macrophotographs confirm the effectiveness of the foaming process used and the uniform distribution of PCM in the geopolymer matrix, and also allow for a visual assessment of the impact of fraction size and PCM content on the porous structure—variants with smaller fractions have denser, finer pores, while larger fractions lead to a more open, macroporous structure.

### 3.5. Microscopic Images of Geopolymer Foams with PCM Based on Diatomite and Paraffin

**Figure 6:**
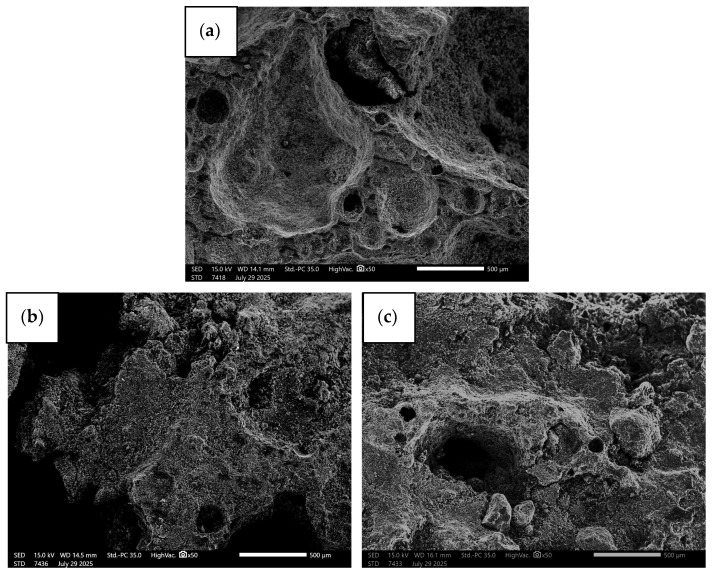

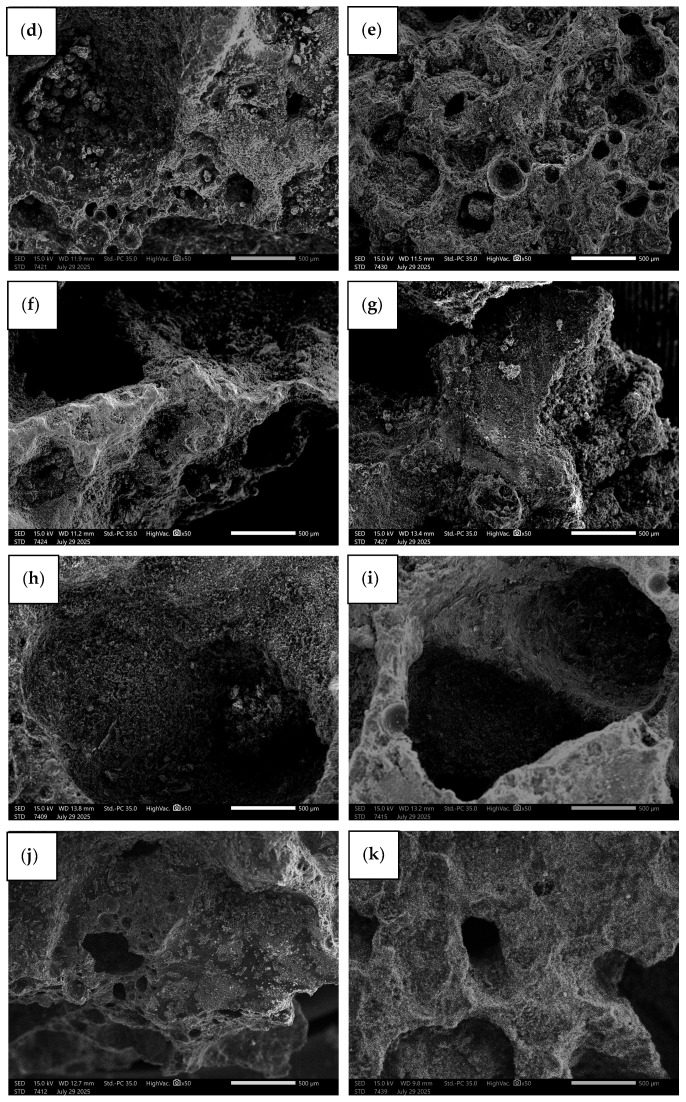
Microscopic images of geopolymer composites with PCMs: (**a**) F.A.—reference, (**b**) <1.6 mm_5 wt.%, (**c**) <1.6 mm_10 wt.%, (**d**) 1.6–1.8 mm_5 wt.%, (**e**) 1.6–1.8 mm_10 wt.%, (**f**) 1.8–2.0 mm_5 wt.%, (**g**) 1.8–2.0 mm_10 wt.%, (**h**) 2.0–2.5 mm_5 wt.%, (**i**) 2.0–2.5 mm_10 wt.%, (**j**) >2.5 mm_5 wt.%, (**k**) >2.5 mm_10 wt.%.

Figure 6 shows microphotographs of the geopolymer composites produced, taken at 50× magnification. The photographs allow for a detailed assessment of the microstructure of the materials, revealing the distribution and shape of the pores and the dispersion of additives in the geopolymer matrix. This makes it possible to visually examine the homogeneity of the geopolymer phase, the presence of any defects, and the impact of the components used on the microstructure of the composite. Such magnification also allows for the analysis of interactions between the additive granules and the matrix, which is important for understanding the mechanical and thermal properties of the materials produced.

Microscopic images of geopolymers with PCM additives show clear correlations between PCM particle size, their content, and the microstructure of the matrix. The reference sample (F.A.) is characterized by a homogeneous, dense structure typical of geopolymers, without visible inclusions or micro-breaks, which serves as a reference point for further analyses. In samples with the smallest PCM particles (<1.6 mm) and low content (5 wt.%), the material shows good particle dispersion in the matrix, with minimal microspaces around the PCM. An increase in content to 10 wt.% causes the appearance of small agglomerates and micro-gaps around some particles, which may indicate limited compatibility of PCM with the matrix. In the case of samples with particles 1.6–1.8 mm in diameter and 5 wt.% PCM, larger particles are still well embedded in the matrix, although the structure becomes less homogeneous. At 10 wt.% PCM, more pronounced cracks are observed around the edges of the particles, which may be the result of the thermal effect of PCM during geopolymer bonding. A further increase in particle diameter to 1.8–2.0 mm causes an increase in porosity around the particles, and at a content of 10 wt.%, more pronounced micro-gaps and local agglomerations appear, which can negatively affect mechanical properties. Samples with particles of 2.0–2.5 mm already show a much more heterogeneous microstructure. At 5 wt.% PCM, there are single cracks in the PCM–matrix contact zones, while at 10 wt%, particle agglomerations and microspaces are observed, indicating limited integration of PCM with the geopolymer. In samples with the largest particles (>2.5 mm), even at 5 wt.% PCM, local structural defects, and distinct pores around the particles appear. At 10 wt.% PCM, the microstructure becomes the most heterogeneous, with numerous microvoids and agglomerates, which may lead to a weakening of mechanical properties, despite a potential increase in heat storage capacity. In general, it can be concluded that as both the diameter of the PCM particles and their content in the composite increase, the microstructure becomes less and less homogeneous, and the number of microvoids and agglomerates increases. The best dispersion and the least impact on the integrity of the matrix are exhibited by the smallest PCM particles at low content, while larger particles and higher PCM concentrations promote the formation of structural defects, which may affect the strength of the geopolymer.

## 4. Discussion

The obtained thermal conductivity parameters λ (≈0.090–0.128 W/m·K) and apparent density (≈338–550 kg/m^3^) are within the range characteristic of lightweight geopolymer foams and foam concretes. In this type of material, a typical correlation between density and heat transfer and mechanical properties is observed—a reduction in density resulting from an increase in porosity leads to a decrease in thermal conductivity, but at the same time results in a decrease in mechanical strength, especially compressive strength [80,81,82]. This classic relationship is also clearly visible in the tests carried out. The sample with a fraction of 1.6–1.8 mm and an additive content of 10 wt.% (ρ ≈ 550 kg/m^3^) is characterized by the highest thermal conductivity λ = 0.12883 W/m· K and the lowest thermal resistance R = 0.2021 m^2^·K/W. This result indicates a more compact microstructure of the composite and a limited proportion of pores with an insulating function. In turn, variants based on fractions of 2.0–2.5 mm_5 wt.% and >2.5 mm_5 wt.% show densities similar to the reference sample or only slightly higher, which translates into the lowest thermal conductivity values (λ = 0.09027–0.09123 W/m·K) and the highest thermal resistances (R ≈ 0.287–0.289 m^2^·K/W). These results are consistent with the literature, which repeatedly emphasizes the quasi-linear relationship between thermal conductivity and matrix density and the proportion of solid bridges in the microstructure of porous materials. Similar observations have been reported in numerous studies devoted to foam concrete and porous geopolymers [80,82,83,84].

The addition of PCM granules to the tested composites leads to an increase in their specific heat capacity (Cp), which clearly confirms the accumulation function of this type of additive. The Cp value of the reference material, which is 1.280 kJ/kg·K, increases to a maximum of 1.873 kJ/kg·K for the variant containing 10 wt.% of the 2.0–2.5 mm fraction. The results obtained are consistent with reports in the literature, which show that the implementation of paraffin phase change materials (PCMs), both in pure form and immobilized on mineral carriers such as silica or diatomite, increases the thermal capacity of composites and enables effective mitigation of temperature fluctuations [85,86,87,88,89]. At the same time, the effect of PCM addition on thermal conductivity (λ) is not clear. Many studies indicate that a moderate proportion of PCMs and appropriately selected grain size may not cause a deterioration in thermal conductivity, and in some cases may even lead to its reduction. This is because the presence of PCM granules may promote the stabilization of the structure with an increased number of closed and fine pores, which perform an insulating function. On the other hand, excessive dosing of fine PCM fractions or particle agglomeration can create additional conduction bridges in the matrix and thus contribute to an increase in λ [82,85,90]. In the analyzed samples, the most favorable compromise between insulation and accumulation properties was observed for variants with a fraction of 2.0–2.5 mm in an amount of 5–10 wt.%. These materials were characterized by low λ values with a simultaneous increase in Cp. The result obtained is in line with the general trend reported in the literature, according to which the optimal selection of the size of the carrier and the amount of PCM is a critical factor for simultaneously minimizing thermal conductivity and maximizing thermal energy storage capacity [85,86,87,89,91].

Macrophotography analysis revealed that the composites under investigation are characterized by diverse pore morphology, including both closed and partially interconnected pores. This type of mixed microstructure determines, on the one hand, favorable insulating properties (due to the presence of closed pores) and, on the other hand, enables partial distribution of mechanical loads through fixed bridges. The 1.6–1.8 mm_10 wt.% variant achieved the highest compressive strength of 3.1 MPa, which correlates closely with its increased density and more compact, rigid matrix. The range of strengths obtained (0.7–3.1 MPa) is typical for lightweight geopolymer foams, in which the thermal conductivity is within the range of λ ≈ 0.09–0.13 W/m·K [80,81,84]. These data confirm a well-documented relationship in the literature: an increase in porosity leads to improved thermal insulation properties (lower λ), but at the same time weakens the mechanical properties of the material by reducing the continuity of the solid phase. Optimization of the foaming process parameters, as well as control of granulation and additive content, allows for a conscious compromise between thermal insulation and mechanical load-bearing capacity, which makes it possible to adapt the characteristics of the material to specific application requirements [80,81,84].

The increase in the total volume of intruded pores and the increase in specific surface area—observed in some variants with PCM addition—indicate a shift in pore distribution towards micropores and fine mesopores. This type of microstructure promotes a reduction in thermal conductivity λ, while maintaining adequate continuity of the solid phase in the matrix. However, it is important to emphasize the limitations of the mercury porosimetry (MIP) technique, in particular the so-called “bottleneck” effect and measurement errors associated with the presence of closed pores. For this reason, correlation and verification of the obtained data using macroscopic imaging and SEM methods, as well as reference to physicothermic parameters (λ–R–ρ), constitute an appropriate and recommended approach [92,93,94]. In the tested samples, good agreement was observed between the trends obtained from the MIP analysis and the macrographic observations, which revealed a more uniform and finer porous structure in the variants with a fraction of 2.0–2.5 mm. These results support the conclusion that the key factor determining the thermal insulation and mechanical properties is the morphology of the pores, and not only the quantitative share of PCM in the matrix.

If the main design criterion is to maximize thermal energy storage (TES) properties, the most promising variants appear to be those with a fraction of 2.0–2.5 mm and a PCM content of 5–10 wt.%, which are characterized by low thermal conductivity λ, high thermal resistance R, increased heat capacity Cp, and moderate density ρ. However, in applications where increased mechanical stiffness is a priority at the expense of insulating properties, the 1.6–1.8 mm_10 wt.% variant provides the highest compressive strength Rc, but at the same time shows an increase in λ. The literature indicates that further optimization of the connections between PCM and the matrix—for example, through the functionalization of diatomite carriers with nanofillers or the addition of graphite—can effectively limit the increase in thermal conductivity while maintaining high heat capacity Cp [86,87,89,91].

## 5. Conclusions

Based on the research conducted, the following conclusions can be drawn. The tested lightweight geopolymer composites with the addition of paraffin phase change materials (PCMs) showed the possibility of achieving an optimal compromise between thermal insulation and energy storage capacity. The most favorable properties were obtained for variants with medium-sized granules and moderate PCM content, which combined low thermal conductivity, high thermal resistance, increased heat capacity, and moderate density, making them the most effective for applications requiring simultaneous insulation and heat accumulation. In turn, samples with fine granules and a higher PCM content achieved the highest compressive strength, but at the expense of deteriorating insulation properties due to increased thermal conductivity, confirming the expected compromise between porosity and the mechanical and thermal properties of lightweight geopolymer foams.

MIP analyses and macro- and SEM observations have shown that the shape and distribution of pores, including the proportion of closed pores and fine mesopores, play a key role in shaping mechanical and thermal properties, regardless of the PCM content itself. The addition of PCM increases the heat capacity of composites, while its effect on thermal conductivity is more complex. Optimal granule size and moderate PCM content allow for reduced conductivity while maintaining high heat capacity, whereas excessive use of fine fractions can lead to increased conductivity through the formation of additional conductive bridges.

Further optimization of PCM connections with the matrix, for example, through the functionalization of diatomite carriers with nanoadditives or the introduction of graphite, is a promising direction for increasing insulation and accumulation properties while limiting the increase in thermal conductivity and maintaining high heat capacity. The results of the research clearly indicate that conscious control of the size of the PCM fraction, its content, and the microstructure of the pores enables the design of lightweight geopolymer composites with properties tailored to specific applications—from insulating materials with heat storage functions to composites with higher mechanical strength.

The limitations of this study stem mainly from the scope of the materials and methodology used. The analysis covered only specific PCM contents and selected granule fractions, and only compressive strength was evaluated, without testing other mechanical properties and long-term durability. The effect of temperature cycles and the stability of PCMs during repeated heating and cooling, which may be important in actual use conditions, were also not taken into account. Furthermore, the tests were conducted on laboratory samples, so the behavior of the material in full-scale structural elements may differ. Finally, the effect of additional modifications to the geopolymer matrix composition was not analyzed, which limits the possibility of directly generalizing the results to other composite variants. In future studies, it would be worthwhile to expand the scope of the analyses to include, among other things, the effect of higher and lower PCM contents, different particle sizes, and other types of phase change materials. It will also be important to investigate additional mechanical properties and to evaluate the long-term durability and behavior of the material under repeated temperature cycles. Furthermore, subsequent work could include testing the material on a scale of actual structural elements and experimenting with modifications to the geopolymer matrix to optimize both its insulating and mechanical properties.

## Figures and Tables

**Table 1:** Oxide analysis for raw materials used for the production of geopolymer foams [71].

Precursor		Oxide Composition (wt.%)					
	SiO_2_	Al_2_O_3_	Fe_2_O_3_	CaO	K_2_O	TiO_2_	SO_3_
**Fly ash**	59.21	31.05	3.88	2.29	2.07	0.77	0.50
**Sand**	98.57	-	0.30	0.36	0.42	-	0.24
**Cement**	-	82.85	-	17.09	-	-	-
**Microspheres**	55.38	38.22	2.44	0.67	2.15	0.85	0.12

**Table 2:** Particle size analysis of raw materials used for the production of geopolymer foams [71].

Material	D_10_ [μm]	D_50_ [μm]	D_90_ [μm]	Average Value [μm]	Standard Deviation [μm]
**Fly ash**	2.45	12.87	32.12	16.21	0.03
**Sand**	253.72	341.97	472.38	390.65	0.02
**Cement**	1.62	11.39	29.90	14.70	0.01
**Microspheres**	18.93	53.39	88.89	56.39	0.07
**Syringaldehyde**	3.04	3.78	7.24	5.07	0.21

**Table 3:** Thermal parameters of diatomite-paraffin granules.

Dimensions of Granules	λ at 0–20 °C [W/m × K]	R at 0–20 °C [m^2^ × K/W]	Cp at 27.5–32.5 °C [kJ/kg × K]
**<1.6 mm**	0.12070	0.2579	1.536
**1.6–1.8 mm**	0.11147	0.2355	1.435
**1.8–2.0 mm**	0.10562	0.2378	1.548
**2.0–2.5 mm**	0.11030	0.2234	1.160
**>2.5 mm**	0.11838	0.2485	1.550

**Table 4:** Material characteristics and markings of geopolymer compositions.

Sample ID	Sand [g]	Microspheres [g]	Fly Ash [g]	Cement [g]	Stabilizer [g]	PCM [g]	H_2_O_2_ [mL]	Alkaline Activator [mL]
F.A.—reference	80	160	795	100	5	0	25	350
<1.6 mm_5 wt.%	80	160	795	100	5	57	25	365
<1.6 mm_10 wt.%	80	160	795	100	5	114	25	370
1.6–1.8 mm_5 wt.%	80	160	795	100	5	57	25	370
1.6–1.8 mm_10 wt.%	80	160	795	100	5	114	25	370
1.8–2.0 mm_5 wt.%	80	160	795	100	5	57	25	370
1.8–2.0 mm_10 wt.%	80	160	795	100	5	114	25	390
2.0–2.5 mm_5 wt.%	80	160	795	100	5	57	25	400
2.0–2.5 mm_10 wt.%	80	160	795	100	5	114	25	400
>2.5 mm_5 wt.%	80	160	795	100	5	57	25	400
>2.5 mm_10 wt.%	80	160	795	100	5	114	25	400

**Table 5:** Porosity of geopolymer foams loaded with diatomite-paraffin granules.

Material	Total Porosity [%]	Pore Diameter Range [μm]	Total Surface Area [m^2^/g]	Total Intruded Volume [cm^3^/g]
**F.A.—reference**	6.60	4.26–210.43	0.0574	0.2642
**<1.6 mm_5 wt.%**	13.47 ↑104.1%	4.26–257.29	0.0598 ↑4.2%	0.4345 ↑64.5%
**<1.6 mm_10 wt.%**	15.44 ↑133.9%	4.26–235.12	0.0521 ↓9.2%	0.3765 ↑42.5%
**1.6–1.8 mm_5 wt.%**	12.81↑94.1%	4.26–210.43	0.0549 ↓4.4%	0.3767 ↑42.6%
**1.6–1.8 mm_10 wt.%**	19.64 ↑197.6%	4.26–235.12	0.0457 ↓20.4%	0.4676 ↑77.0%
**1.8–2.0 mm_5 wt.%**	18.44 ↑179.4%	4.27–229.65	0.0573 ↓0.2%	0.4192 ↑58.7%
**1.8–2.0 mm_10 wt.%**	13.29 ↑101.4%	4.27–229.65	0.0451 ↓21.4%	0.3408 ↑29.0%
**2.0–2.5 mm_5 wt.%**	5.38 ↓18.5%	4.26–195.67	0.0307 ↓46.5%	0.1793 ↓32.1%
**2.0–2.5 mm_10 wt.%**	12.28 ↑86.1%	4.27–246.42	0.0366 ↓36.2%	0.2995 ↑13.4%
**>2.5 mm_5 wt.%**	9.60 ↑45.5%	4.27–246.42	0.0430 ↓25.1%	0.3098 ↑17.3%
**>2.5 mm_10 wt.%**	14.26 ↑116.1%	4.26–257.29	0.0531 ↓7.5%	0.4195 ↑58.8%

Green color—increase. Red color—decrease.

**Table 6:** Thermophysical parameters of geopolymer foams loaded with diatomite-paraffin granules.

Material	Density [kg/m^3^]	λ at 0–20 °C [W/m × K]	R at 0–20 °C [m^2^ × K/W]	Cp at 27.5–32.5 °C [kJ/kg × K]
**F.A.—reference**	337.65	0.09248	0.2831	1.280
**<1.6 mm_5 wt.%**	377.80 ↑11.92%	0.09472 ↑2.40%	0.2851 ↑0.71%	1.560 ↑21.88%
**<1.6 mm_10 wt.%**	406.45 ↑20.36%	0.09813 ↑6.12%	0.2684 ↓5.21%	1.587 ↑23.83%
**1.6–1.8 mm_5 wt.%**	379.70 ↑12.50%	0.09440 ↑2.08%	0.2722 ↓3.88%	1.692 ↑32.19%
**1.6–1.8 mm_10 wt.%**	550.00 ↑62.90%	0.12883 ↑39.18%	0.2021 ↓28.61%	1.552 ↑21.25%
**1.8–2.0 mm_5 wt.%**	434.25 ↑28.63%	0.09790 ↑5.84%	0.2631 ↓7.09%	1.813 ↑41.64%
**1.8–2.0 mm_10 wt.%**	416.40 ↑23.34%	0.10141 ↑9.68%	0.2569 ↓9.27%	1.648 ↑28.75%
**2.0–2.5 mm_5 wt.%**	339.20 ↑0.44%	0.09027 ↓2.39%	0.2888 ↑1.91%	1.691 ↑32.23%
**2.0–2.5 mm_10 wt.%**	368.95 ↑9.29%	0.09533 ↑3.06%	0.2729 ↓3.61%	1.873 ↑46.33%
**>2.5 mm_5 wt.%**	378.40 ↑12.11%	0.09123 ↓1.36%	0.2867 ↑1.22%	1.682 ↑31.56%
**>2.5 mm_10 wt.%**	435.85 ↑29.14%	0.10392 ↑12.36%	0.2524 ↓10.86%	1.488 ↑16.25%

Green color—increase. Red color—decrease.

## Data Availability

The original contributions presented in this study are included in the article. Further inquiries can be directed to the author.
